# No big data without small data: learning health care systems begin and end with the individual patient

**DOI:** 10.1111/jep.12350

**Published:** 2015-03-31

**Authors:** José A. Sacristán, Tatiana Dilla

**Affiliations:** ^1^ Medical Department Lilly Madrid Spain

## Introduction

We live in the era of big data. Data volume doubles every 2 years and it has been estimated that every 2 days, more data are generated than were produced in human history up to 2003. The development of technology and new analytical capabilities, which allow the handling of large data volumes from different sources, have generated high expectations regarding the potential of big data for understanding the world and aiding in decision making [1]. Technological development is so rapid that it is difficult to imagine all the applications that may result from the analyses of the data that are generated globally every day.

A broad consensus exists concerning the vast possibilities of big data in research and in the optimization of medical care, improving their quality and reducing their cost [2, 3]. However, big data applications in the health sector lag behind those of other areas of knowledge, such as the physical sciences, economics, businesses or social networks, where data mining techniques are giving rise to unprecedented qualitative changes [4].

Variability is the essence of biomedical sciences. In medicine, the heterogeneity of individuals calls for personalized decisions to benefit individual patients. Theoretically, the potential of big data in the field of health may be limited by increasingly personalized medicine. This paper analyses the potential barriers that may impede the development of big data in medicine and research and proposes ways of moving forward to generate a ‘learning health care system’ that aims to improve health outcomes for current and future patients in an efficient manner.

## Barriers that may slow the development of big data in research and medicine

The main limitations of big data in clinical research and in medical care are well known and are related to methodological, technologic and legal factors. Among the methodological barriers, the low quality of data (incomplete data, lack of standardization) and the existence of an analytical methodology that remains insufficiently developed are most prominent [5, 6]. The biases inherent to the analyses conducted on databases (often used for administrative and billing purposes) have been widely described [7]. Obvious technical and analytical difficulties exist in managing a very large volume of data that is constantly changing and that resides in different repositories, along with frequent linkage and interoperability issues. A significant part of the data is ‘noise’, which presents challenges when the noise grows faster than the signal. Different databases with different degrees of quality and completeness generate heterogeneous results [8, 9], which may increase the risk of ‘biased fact‐finding excursions’ and false discoveries [5]. Finally, restrictions in access to databases and privacy problems are generating growing concern among experts [10].

However, although the barriers mentioned earlier are important, a problem of even greater significance is hindering the application of big data in medicine. In the era of personalized medicine, the real challenge of big data is *how to use large‐scale population‐based analyses to benefit individual patients*. This situation reflects the classical conflict between the objectives of clinical research and those of medical care. The purpose of clinical research is to generate generalizable knowledge that is useful for future patients, whereas medical care aims to promote the well‐being of individual patients. Whereas clinical research seeks generalization, medical care seeks personalization.

The previous conflict is exhibited in the two most important movements that have emerged in health systems in the last decades: Evidence‐based medicine and patient‐centred medicine. Evidence‐based medicine has its conceptual anchor in research, valuing evidence that results from experimental methods such as the randomized clinical trial (RCT), particularly when such trials involve large sample sizes. The primary objective of evidence‐based medicine is generalization and development of clinical guidelines and the standardization of medical care. In contrast, patient‐centred medicine has its conceptual anchor in medical care; therefore, its reference is the individual patient, a patient whose personal beliefs, objectives and preferences are, essentially, unique and non‐transferable [11].

## The doctor–patient encounter as the link between clinical research and medical care

**Figure 1:**
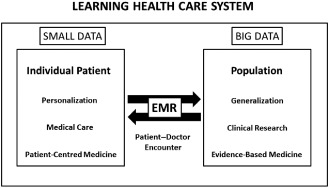
Learning health care system. Each medical act is the intersection between the small and big data.

The worlds of populations and individuals must necessarily converge in the path that separates evidence‐based medicine and patient‐centred medicine. Every doctor–patient encounter represents the connecting link between the population and the individual, between clinical research and medical care [2]. The progressive implementation of electronic medical records (EMRs) may help to gradually blur the borders between research and care, contributing to the creation of a true ‘rapid‐learning health system’ [12] in which each medical act has the double objective of generating and applying clinically relevant medical knowledge. This approach should produce benefits for present and future patients as follows: (1) the data generated in each medical act should not only be used to the benefit of that individual patient but also to generate knowledge potentially useful for future patients and (2) all of the knowledge generated through big data analyses should be applied to improve clinical decision making and to produce benefits for individual patients. Neither of these two goals is achieved at present: most of the information generated in each medical act is lost and it requires an estimated average of 17 years for only 14% of new discoveries to enter into daily clinical practice [13]. In summary, the individual and the population and the small data and the big data are the two sides of the same coin and EMRs are the intersection between small data (individual data, relatively easy to handle, used by the doctor in his/her consultation to personalize medical care) and big data (Fig. 1).

**Figure 2:**
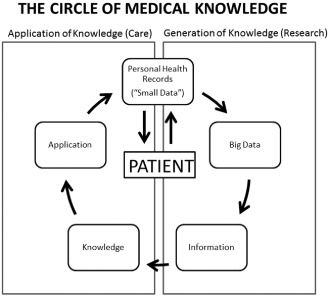
The circle of medical knowledge begins and ends with the individual patient.

Figure 2 presents the ‘circle of knowledge’ and describes how clinical research and medical care begin and end with the individual patient. Each doctor–patient encounter generates data (small data), which are collected in the EMR. The sum of millions of small data gives rise to big data, which should be analysed and translated into information. The information is only useful if it is translated into knowledge and *knowledge is only useful if it is used to improve the health of individual patients*. One of the main reasons why big data have not fulfilled its full potential in health care is that small data are not adequately systematized to generate useful knowledge for future patients (research) and that big data are not used to improve health outcomes for individual patients (care). In the following sections, proposals are offered to attempt to solve these challenges as follows: (1) how to use small data to generate knowledge and (2) how to use big data to benefit individual patients.

## How to use ‘small data’ to generate knowledge

Although low data quality and biases because of the absence of randomization are two of the main limitations of observational research, most efforts to increase the value of big data are oriented towards analysing all of the existing data through the linkage of different databases. Interestingly, the obsession of modern science for measurement (and biomedicine is no exception) has contributed to the transformation of tools into goals, promoting the idea that everything that can be measured must be measured and identifying quantity with quality and big data with ‘reliable data’. Because massive amounts of data and very powerful technology exist, we have fallen into ‘data‐ism’ [14].

Initiatives to analyse non‐structured data (e.g. the analysis of free text through natural language processing and pattern recognition) are more than welcome, although they are insufficient. The value of big data will be greatly limited if the data are not based on high‐quality individual clinical data and structured formats. For that reason, it appears reasonable to redefine the present priorities, devoting greater efforts towards generating standardized and complete data that include both quantitative clinical variables as well as qualitative impressions of the doctors and the preferences and important variables of the patients [15].

The lack of a control group and the absence of randomization are other important limitations of observational data, particularly when the objective is to assess the effectiveness of health interventions and to predict outcomes [6]. One way of overcoming these limitations is to integrate investigational efforts into clinical practice to conduct point of care research. For example, patients treated under conditions of typical clinical practice who met the specific pre‐determined selection criteria might be automatically identified to participate in randomized registry trials. The idea of conducting ‘randomized database studies’ combining the advantages of initial randomization (minimization of biases) and the advantages of the follow‐up of patients treated under routine clinical practice was described for the first time in 1998 [16]. This proposal, which has been recently been considered as the next revolution in clinical research [17], has been successfully implemented in recent years [18, 19] because of the technical developments of EMRs that allow the embedding of trials into regular medical practice. The United States National Institutes of Health is designing and conducting several pragmatic trials that exploit routinely collected data, including patient‐reported outcomes, to quickly demonstrate effectiveness in real‐world care delivery systems [2]. Another way to integrate experiments into clinical practice would be to conduct N‐of‐1 trials. This form of research has only been rarely applied despite its clear advantages in benefitting the individual patients who participate in these studies [20].

The integration of experiments in daily practice requires important regulatory and cultural changes oriented towards decreasing the level of regulatory oversight and adapting the ethical requirements to the risk for the patients. Recently, prominent bioethicists have suggested that informed consent documents could be substantially simplified (or even suppressed) in the case of pragmatic trials that assess established interventions for which there are minimal incremental risks and burdens compared with usual clinical care (e.g. a comparative effectiveness study that compares two standard‐of‐care interventions) [21, 22]. In these ‘low risk’ conditions, the informed consent document could be similar to the simple consent document used in clinical practice, as the main distinguishing feature of these trials is that they replace clinical uncertainty with randomization [23].

To eliminate the cultural barriers that exist between clinical research and medical care, it may be useful to realize that all research begins at the patient's bedside and that every medical act is structured similar to an experiment. The increasing use of EMRs might contribute to the elimination of walls between doctors who conduct research and those who do not, between patients who participate in RCTs and the ‘real’ patients who doctors see every day and between the clinical research form used in RCTs and the electronic medical history. In all likelihood, the real challenge to embedding research into daily clinical practice is not the technical infrastructure to implement randomized database studies but the understanding that, in the context of learning health care systems, a clear distinction between research and care should not exist [24].

## How to use big data to benefit individual patients

Among all of the activities that have successfully applied the analyses of big data, ‘the key has been to go beyond aggregate data and link information to individual people’ [25]. In health care, big data analyses have not been systematically transformed into benefits for individual patients. This is likely the main reason why the use of big data in medicine is relatively delayed compared with other fields of knowledge. Once new knowledge has been generated, it is essential to apply it to aid doctors in their daily practice in an individualized and rapid manner. EMRs will become much more valuable tools for doctors if they are designed for use in personalized medical care.

There are several potential ways of applying the knowledge resulting from big data at the individual level. Perhaps, the most obvious method is the use of decision‐aid systems that help doctors in the diagnosis and treatment of their patients. Many of the present tools are linked to evidence‐based guidelines and recommendations. However, very often, these guidelines are based on the results of large RCTs and meta‐analyses containing information that, in theory, is applicable to ‘average patients’. However, doctors do not treat average patients; thus, this ‘generalizable knowledge’ that may be useful to standardize medical practice is not the most appropriate to treat individual patients. Currently, the real challenge is to develop more ‘personalized guidelines’ that take into account the heterogeneity of patients and aid doctors in individualizing their clinical decisions [26]. Fortunately, new guidelines increasingly include tailored recommendations for subgroups of patients and examples are evident wherein the preferences and values of individual patients are the key drivers for the recommendation [27].

EMRs could also help doctors identify ‘anomalies’ or unexpected results, test hypotheses and identify possible areas of intervention [25]. For example, predictive analytics may identify situations in which a given patient exhibits a high risk of complications or may detect the existence of characteristics that could predict a certain behaviour (e.g. risk of low compliance to treatment, high risk of adverse events or readmission) [28]. Patient support programmes could be linked to EMRs to help doctors handle particularly complex situations and optimize medical care.

EMRs may also be used to engage patients, facilitating shared decision‐making processes [29] and more active participation of patients in clinical research. For example, EMRs could be linked to ‘patient decision aids’ designed to help patients better understand the benefits and risks of different alternatives and aid them in reflecting on the pros and cons of the different options [30]. With respect to clinical research, EMRs could include information about the selection criteria of ongoing clinical trials and information on the participant centres so that doctors might offer patients the possibility of being candidates for such trials. Finally, EMRs could contribute to adapting the level of information and regulatory oversight to the individual characteristics and cultural level of each patient. For example, the informed consent document could be adapted to both the level of risk of the study and the literacy of each patient. In the same way, the system could provide tailored information on the results of the study at a level of complexity adapted to the needs of the patient.

## Summary

The apparent contradiction between the population focus of big data and the practice of personalized medicine contributes to the relatively scarce and slow applications of big data in medicine compared with other areas of knowledge. The technologic development and the implementation of EMRs may give rise to a learning health care system in which every doctor–patient encounter becomes the connecting link between the population and the individual.

To generate valuable knowledge, big data must come from high‐quality individual clinical data. *There are no big data without small data*. EMRs may be used to integrate research into medical care, thereby conducting point of care research (e.g. *randomized database studies)*. However, big data will not achieve its full potential if it is not used to improve health outcomes for the individual patients from whom the data were generated.

EMRs should aid doctors in personalizing medical care and contribute towards the engagement of patients in research and care. The continuous interaction between the individual patient and the population, between clinical research and medical care, between the world of big data and that of small data is an essential step towards achieving a true learning health care system.

## Conflict of interest

José A. Sacristán and Tatiana Dilla are employees of Lilly. Any views or opinions presented in this manuscript are solely those of the authors and do not necessarily represent those of Lilly.

## Author contributions

José A. Sacristán developed the concept and design of this manuscript and drafted article. Tatiana Dilla collaborated in the acquisition of information for the manuscript. José A. Sacristán and Tatiana Dilla both participated in the critical revision of the article and its final approval.

## References

[1] Murdoch, T. B. & Detsky, A. S. (2013) The inevitable application of big data to health care. Journal of the American Medical Association, 13, 1351–1352. 10.1001/jama.2013.393 23549579

[2] Larson, E. B. (2013) Building trust in the power of ‘big data’ research to serve the public good. Journal of the American Medical Association, 309, 2443–2444. 23780455 10.1001/jama.2013.5914 PMC12128613

[3] Roski, J. , Bo‐Linn, G. W. & Andrews, T. A. (2014) Creating value in health care through big data: opportunities and policy implications. Health Affairs, 33, 1115–1122. 25006136 10.1377/hlthaff.2014.0147

[4] Davenport, T. H. & Harris, J. G. (2007) Competing on Analytics: The New Science of Winning. Boston, MA: Harvard Business School Press.

[5] Wang, W. & Krishnan, E. (2014) Big data and clinicians: a review on the state of the science. Journal of Medical Internet Research Medical Informatics, 2, 1–11. 10.2196/medinform.2913 PMC4288113 25600256

[6] Schneeweiss, S. (2014) Learning from big health care data. The New England Journal of Medicine, 370, 2161–2163. 24897079 10.1056/NEJMp1401111

[7] Schneeweiss, S. & Avorn, J. (2005) A review of uses of health care utilization databases for epidemiologic research on therapeutics. Journal of Clinical Epidemiology, 58, 323–337. 15862718 10.1016/j.jclinepi.2004.10.012

[8] Madigan, D. , Ryan, P. B. , Schuemie, M. , Stang, P. E. , Overhage, J. M. , Hartzema, A. G. , Suchard, M. A. , DuMouchel, W. & Berlin, J. A. (2013) Evaluating the impact of database heterogeneity on observational study results. American Journal of Epidemiology, 178, 645–651. 23648805 10.1093/aje/kwt010 PMC3736754

[9] Psaty, B. M. & Breckenridge, A. M. (2014) Mini‐Sentinel and regulatory science. Big data rendered fit and functional. The New England Journal of Medicine, 370 (23), 2165–2167. 24897081 10.1056/NEJMp1401664

[10] Ross, J. S. & Krumholz, H. M. (2013) Ushering in a new era of open science through data sharing. The wall must come down. Journal of the American Medical Association, 309, 1355–1356. 23508736 10.1001/jama.2013.1299

[11] Sacristan, J. A. (2013) Evidence based medicine and patient centered medicine: some thoughts on their integration. Revista Clínica Española, 213, 460–464. 24409523

[12] Etheredge, M. L. (2007) A rapid‐learning health system. Health Affairs, 26, w107–w118. 17259191 10.1377/hlthaff.26.2.w107

[13] Balas, E. A. & Boren, S. A. (2000) Yearbook of Medical Informatics: Managing Clinical Knowledge for Health Care Improvement. Stuttgart, Germany: Schattauer Verlagsgesellscaft mbH. 27699347

[14] Brooks, D. (2013) The philosophy of data. New York Times, 4 Feb.

[15] Black, N. (2013) Patient reported outcome measures could help transform healthcare. British Medical Journal, 346, f167. 23358487 10.1136/bmj.f167

[16] Sacristan, J. A. , Soto, J. , Galende, I. & Hylan, T. R. (1998) Randomized database studies: a new method to assess drugs' effectiveness? Journal of Clinical Epidemiology, 51, 713–715. 9731918 10.1016/s0895-4356(98)00058-4

[17] Lauer, M. S. & D'Agostino, R. B. (2013) The randomized registry trial. The next disruptive technology in clinical research? The New England Journal of Medicine, 369 (17), 1579–1581. 23991657 10.1056/NEJMp1310102

[18] Staa, T. P. , Goldacre, B. , Gulliford, M. , Cassell, J. , Pirmohamed, M. , Taweel, A. , Delaney, B. & Smeeth, L. (2013) Pragmatic randomised trials using routine electronic health records: putting them to the test. British Medical Journal, 344, e55. 10.1136/bmj.e55 PMC3934788 22315246

[19] van Staa, T. P. , Dyson, L. , McCann, G. , *et al* (2014) The opportunities and challenges of pragmatic point‐of‐care randomised trials using routinely collected electronic records: evaluations of two exemplar trials. Health Technology Assessment, 18, 1–146. 10.3310/hta18430 PMC4781625 25011568

[20] Duan, N. , Kravitz, R. L. & Schmid, C. H. (2013) Single‐patient (n‐of‐1) trials: a pragmatic clinical decision methodology for patient‐centered comparative effectiveness research. Journal of Clinical Epidemiology, 66 (8 Suppl.), S21–S28. 23849149 10.1016/j.jclinepi.2013.04.006 PMC3972259

[21] Kass, N. , Faden, R. & Tunis, S. (2012) Addressing low‐risk comparative effectiveness research in proposed changes to US federal regulations governing research. Journal of the American Medical Association, 307, 1589–1590. 22511685 10.1001/jama.2012.491

[22] Faden, R. R. , Kass, N. E. , Goodman, S. N. , Pronovost, P. , Tunis, S. & Beauchamp, T. L. (2013) An ethics framework for a learning health care system: a departure from traditional research ethics and clinical ethics. Hastings Center Report, 43, S16–S27. 10.1002/hast.134 23315888

[23] Faden, R. R. , Beauchamp, T. L. & Kass, N. E. (2014) Informed consent, comparative effectiveness, and learning health care. The New England Journal of Medicine, 370, 766–768. 24552325 10.1056/NEJMhle1313674

[24] Sacristan, J. A. (2013) Patient‐centered medicine and patient‐oriented research: improving health outcomes for individual patients. BMC Medical Informatics and Decision Making, 13, 6. 23294526 10.1186/1472-6947-13-6 PMC3575265

[25] Weber, G. M. , Mandl, K. D. & Kohane, I. S. (2014) Finding the missing link for big biomedical data. Journal of the American Medical Association, 24, 2479–2480. 10.1001/jama.2014.4228 24854141

[26] Montori, V. M. , Brito, J. P. & Murad, M. H. (2013) The optimal practice of evidence based medicine. Incorporating patient preferences in practice guidelines. Journal of the American Medical Association, 310, 2503–2504. 24165826 10.1001/jama.2013.281422

[27] Hayes, J. H. & Barry, M. J. (2014) Screening for prostate cancer with the prostate‐specific antigen test. A review of current evidence. Journal of the American Medical Association, 311, 1143–1149. 24643604 10.1001/jama.2014.2085

[28] Bates, D. W. , Saria, S. , Ohno‐Machado, L. , Shah, A. & Escobar, G. (2014) Big data in health care: using analytics to identify and manage high‐risk and high‐cost patients. Health Affairs, 33, 1123–1131. 25006137 10.1377/hlthaff.2014.0041

[29] Lipkin, M. (2013) Shared decision making. Journal of the American Medical Association Internal Medicine, 173, 1204–1205. 23712307 10.1001/jamainternmed.2013.6248

[30] Hoffmann, T. C. , Legare, F. , Simmons, M. B. , McNamara, K. , McCaffery, K. , Trevena, L. J. , Hudson, B. , Glasziou, P. P. & Del Mar, C. B. (2014) Shared decision making: what do clinicians need to know and why should they bother? Medical Journal of Australia, 201, 35–39. 24999896 10.5694/mja14.00002

